# Updates on *Parasitology* and adopting a Gold Open Access model of production

**DOI:** 10.1017/S0031182022001329

**Published:** 2022-10

**Authors:** J. R. Stothard, J. T. Ellis

**Affiliations:** 1 Department of Tropical Disease Biology, Liverpool School of Tropical Medicine, Liverpool L3 5QA, UK; 2 School of Life Sciences, University of Technology Sydney, Ultimo, NSW 2007, Australia

## Abstract

**Figure:**
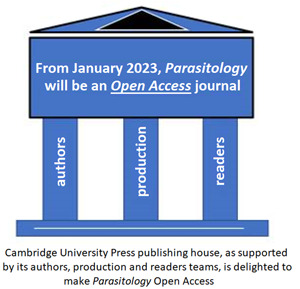

Many readers will have noted that all published articles within *Parasitology* from January 2022 onwards were available online only. The hardcopy production, like many other academic journals, has now ceased. This is true not only for regular issues but also for forthcoming special issues. Moreover, from January 2023, *Parasitology* will become open access (OA) where we adopt a Gold Open Access model, specifically a non-exclusive Gold Open Access CC-BY licence (see https://www.cambridge.org/core/services/open-access-policies/open-access-resources/creative-commons-licenses). This arises from the most appropriate OA choice by Cambridge University Press. The change is not trivial for our current financial plan, primarily based upon institutional subscriptions, is transformed to an article processing charges (APCs) one. In order to accommodate peer review and production lead times, all papers submitted after 3rd October 2022, if accepted, will be published as OA.

By transitioning to OA, *Parasitology* seeks to improve the global accessibility of its articles, with a more equitable international readership, and responds to the behests of research funders requiring authors to publish in OA journals. All submissions to *Parasitology* will continue to be fully peer-reviewed to maintain our high standards. With respect to our on-going attention on research integrity and quality assurance processes, we have adopted the use of *iThenicate* (see https://www.ithenticate.com/) with *Crossref* providing a database of scholarly papers. Using these e-tools, we can better detect any evidence of plagiarism within forthcoming manuscripts once assigned for peer review.

We stress that evidence of plagiarism within our journal is very rare, but these steps are taken from preventive rather than retrospective concerns. Many of you are likely familiar with its sister technology *Turnitin* (see https://www.turnitin.com/) that is regularly used in academic institutions to search for plagiarism in student essays and assessments. Our use of *iThenicate* aligns us with other high-profile publishers such as Springer Nature, Wiley and Elsevier, and also helps us to better assess the novelty of work submitted.

## A regular OA production cycle

Unlike other OA journals that have alternative production of contents, *Parasitology* will maintain its grouping of papers in a monthly production cycle, alongside our ‘Accepted manuscripts’ and ‘FirstView’ online categories. Monthly issues will remain with 12 per year, containing some 8–12 papers, not forgetting our 2 further special issues. The latter are typically commissioned upon specific content themes and are usually published some 6 months apart. Of note, 1 special issue is earmarked for outputs from meeting(s) of the British Society for Parasitology, while the other is upon open commission. Commissions are overseen by Professor John Ellis across a range of contemporary interests in parasites and parasitism.

To broaden *Parasitology*'s international appeal, we encourage interactions with other international societies and agencies aligned with our journal's objectives. To help manage these processes, *Parasitology* has adopted a theory-of-change framework (Stothard *et al*., 2021), where we remain mindful of the many inputs and outputs from a network of both external and internal stakeholders. All of this is vital to maintain the future success of the journal across its many dimensions.

Our activities within the journal's dimensions need to be coordinated, harnessed and then translated into a varied portfolio of academic outputs, outcomes and impacts. For example, the continued help of our academic referees is a particularly important facet. Indeed, we have thanked many experts for their services this past year (see https://www.cambridge.org/core/blog/2022/05/12/a-sincere-thanks-to-our-parasitology-reviewers/) and are considering alternative ways to reward those that give their time *pro bono*.

## Securing a wider international outreach

In the shorter term, *Parasitology* is liaising with one of our editors, Professor Laura Rinaldi, in support of a special issue with the Italian Society for Parasitology (SoIPa) arising from their recent XXXII National Congress held in Naples this year. In the longer term, *Parasitology* will support a future scientific session at the International Congress of Parasitology (ICOPA) 2026 in Canada. The congress arises from cooperative activities of the World Federation of Parasitologists (see https://www.wfpnet.org/) that bring together many learned biological societies across the world.

Furthermore, at their annual meetings, *Parasitology* is proud to continue to sponsor the William C. Campbell postgraduate award of the Irish Society for Parasitology. For those that don't know, William C. Campbell (see https://en.wikipedia.org/wiki/William_C._Campbell_(scientist)) was a joint winner of the 2015 Nobel Prize for Medicine for the discovery of ivermectin. This year Mona Suleiman from the University of Bath was chosen for her presentation and studies on sRNAs in the free-living and parasitic stages of *Strongyloides*. A short blog is soon to appear on our website that embellishes upon her achievements.

Since COVID, there is a well-recognized need for parasitologists to re-initiate face-to-face meetings. These augment discussion of recent knowledge gained and generate livelier and more honest conversations than online fora can. They also better sow the seeds of scientific collaboration and wider public engagement. More details about this and other initiatives within and external to CUP, such as our open image competition for our journal front cover or our Early Career Researcher Award, will follow.

Please continue to view our website regularly and subscribe to our Twitter (@JnlParasitology) and Facebook (https://www.facebook.com/JnlParasitology/) accounts to keep best informed.

## Updates on *Parasitology* during 2022

We have welcomed 2 new academic editors, Professors Cinzia Cantacessi and Joe Jackson, from the Universities of Cambridge and Salford, respectively. They have replaced Professor Andrew Hemphill and Professor Hélène Carabin, each taking over their specialist themes. For just over 10 years, Andrew has had an unfaltering editorial association with the journal, helping to maintain and grow our scientific reputation. We sincerely thank Hélène, who has had to step down to balance an increasing workload at the University of Montreal, for her valued inputs. Over the year, we have witnessed a changing portfolio of increasing submissions about parasites in livestock and wildlife. Thus, Cinzia and Joe will each help to balance these more specialist needs in veterinary and ecological parasitology.

To remind readers, the skill set of each editor is highlighted by the ‘Meet the Editors’ section within our journal's blog. Our general OneHealth interest is maintained across all 7 editors. ‘Meet the Editors’ seeks to help authors and readers better understand our primary editorial skills base and how we might initially triage submitted manuscripts before later peer review. In this online series we also feature the production staff within CUP, who often play, to those unfamiliar with scientific publishing, much underappreciated roles. Since January 2022 our managing editor, Alison Paskins, has been on maternity leave, with Bailey Fallon and latterly Izzy Aaron covering her absence. Ali will soon re-join the *Parasitology* team in early 2023 and in the interim we wish her and her baby boy, Guliuo, much joy.

As our social media and online presence grows, with ‘Paper of the Month’ and commissioned blogs around virtual collections, world awareness days and CUP prizes, we have welcomed Dr Derick Osakunor, Children's National Hospital, USA. Derick joins the social media team of editors, Emily Pascoe and Maureen Williams, with Emily now promoted to Senior Social Media Editor. As authors and readers realize, the use of social media is ever more important in raising general awareness of articles, alongside amplifying their communication and research uptake.

**Fig. 1.**
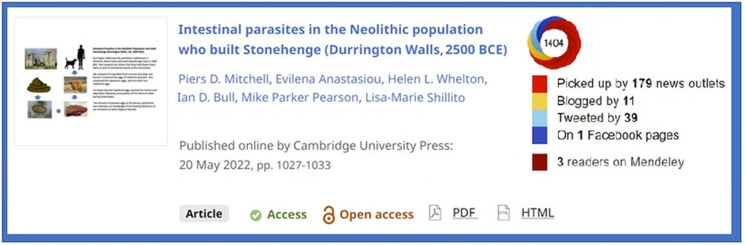
Social media scores of Mitchell *et al*. (2022) demonstrate wider interest in human parasites from the Neolithic period and captures national and international interest. As of 5^th^ September 2022, there were 5467 views of this article with 11 associated blogs, one of which was from New Scientist (see https://www.newscientist.com/article/2321267-the-people-who-built-stonehenge-may-have-eaten-raw-cattle-organs/?utm_campaign=RSS%7CNSNS&utm_source=NSNS&utm_medium=RSS&utm_content=home).

A key social media highlight has been the impressive altmetric scores that Mitchell *et al*. (2022) achieved (Fig. 1). Their article provided a novel insight into the prehistoric past in describing human parasites found in Neolithic populations near Stonehenge. Articles such as this, even though specialist in archaeoparasitology, capture public interest, reminding us that many parasites have quietly, or perhaps, not so quietly, been influencing our recent evolution and civilizations.

## An outlook upon OA and beyond

As OA production has been with us for over 2 decades now, many authors are familiar with the legal requirements and financial implications that APCs require. Nevertheless, there are some authors who are rightly concerned and duly worried about this transition for *Parasitology*. A key question is – *if I have limited funds, how can I afford to publish my articles in OA journals*? Both of us can speak from personal experiences.

For example, JRS is employed by an institute currently not included in the Read and Publish institutional deals with CUP (also known as transformative agreements). While some of his research is supported by The Wellcome Trust, which requires OA publishing, some of his work is not. A perplexing gap therefore remains which, for example, includes how to translate those quality outputs from keen MSc students into published papers. Developing the redacted outputs of those in training should be considered a fundamental right in higher education, yet the playing field to do so remains uneven.

Currently, CUP maintains a database of academic institutes eligible within the Read and Publish agreement (see https://www.cambridge.org/core/services/open-access-policies/read-and-publish-agreements). You can check if your institution is covered by a Read and Publish agreement using the online checker (see https://www.cambridge.org/core/services/open-access-policies/waivers-discounts). If your institute is not included within this list, such as JRS's and JTE's home institutions, then some local canvassing of academic needs is required. Furthermore, CUP has provided additional information about OA in all its many guises (see https://www.cambridge.org/core/services/open-research/open-access/open-access-journal-flips). To reassure our authors, as a supplementary safeguard, we also welcome a pre-submission enquiry to the *editor-in-chief* to help clarify whether an APC waiver could be justified for your article if our existing guiding rubric is unclear.

To close, we look forward to the opportunities that going OA in 2023 might bring. While measures of our future success might recourse to metrics, e.g. impact factors (which has continued to increase, now standing at 3.243), the existence of *Parasitology* is primarily to serve its discipline. Above all, we seek to support those who actively contribute and disseminate the knowledge of parasites and parasitism across the life sciences.

**Editors**

Russell Stothard *Editor-in-Chief*, 2020–

John Ellis *Deputy Editor-in-Chief & Special Issues Editor*, 2021–

Cinzia Cantacessi, 2022–

**Hélène Carabin, 2021–2022**

Joseph Jackson, 2022–

**Andrew Hemphill, 2011–2022**

Laura Rinaldi, 2020–

Lisa Ranford-Cartwright, 2021–

Jonathan Wastling, 2011–

**Social media editors**

Emily Pascoe, 2019–2022, *Senior Social Media Editor*, 2022–

Maureen Williams, 2019–

Derrick Osakunor, 2022–
